# A radiomics model can distinguish solitary pulmonary capillary haemangioma from lung adenocarcinoma

**DOI:** 10.1093/icvts/ivab271

**Published:** 2021-10-14

**Authors:** Hao-Jen Wang, Mong-Wei Lin, Yi-Chang Chen, Li-Wei Chen, Min-Shu Hsieh, Shun-Mao Yang, Ho-Feng Chen, Chuan-Wei Wang, Jin-Shing Chen, Yeun-Chung Chang, Chung-Ming Chen

**Affiliations:** 1 Institute of Biomedical Engineering, College of Medicine and College of Engineering, National Taiwan University, Taipei, Taiwan; 2 Department of Surgery, National Taiwan University Hospital and National Taiwan University College of Medicine, Taipei, Taiwan; 3 Department of Medical Imaging, National Taiwan University Hospital and National Taiwan University College of Medicine, Taipei, Taiwan; 4 Department of Pathology, National Taiwan University Hospital and National Taiwan University College of Medicine, Taipei, Taiwan; 5 Department of Surgery, National Taiwan University Hospital Hsin-Chu Branch, Hsin-Chu City, Taiwan; 6 Department of Surgical Oncology, National Taiwan University Cancer Center, Taipei, Taiwan

**Keywords:** Computed tomography, Ground-glass nodule, Lung adenocarcinoma, Lung cancer surgery, Solitary pulmonary capillary haemangioma

## Abstract

**OBJECTIVES:**

Solitary pulmonary capillary haemangioma (SPCH) is a benign lung tumour that presents as ground-glass nodules on computed tomography (CT) images and mimics lepidic-predominant adenocarcinoma. This study aimed to establish a discriminant model using a radiomic feature analysis to distinguish SPCH from lepidic-predominant adenocarcinoma.

**METHODS:**

In the adenocarcinoma group, all tumours were of the lepidic-predominant subtype with high purity (>70%). A classification model was proposed based on a two-level decision tree and 26 radiomic features extracted from each segmented lesion. For comparison, a baseline model was built with the same 26 features using a support vector machine as the classifier. Both models were assessed by the leave-one-out cross-validation method.

**RESULTS:**

This study included 13 and 49 patients who underwent complete resection for SPCH and adenocarcinoma, respectively. Two sets of features were identified for discrimination between the 2 different histology types. The first set included 2 principal components corresponding to the 2 largest eigenvalues for the root node of the two-level decision tree. The second set comprised 4 selected radiomic features. The area under the receiver operating characteristic curve, accuracy, sensitivity, specificity were 0.954, 91.9%, 92.3% and 91.8% in the proposed classification model, and were 0.805, 85.5%, 61.5% and 91.8% in the baseline model, respectively. The proposed classification model significantly outperformed the baseline model (*P* < 0.05).

**CONCLUSIONS:**

The proposed model could differentiate the 2 different histology types on CT images, and this may help surgeons to preoperatively discriminate SPCH from adenocarcinoma.

**Figure:**
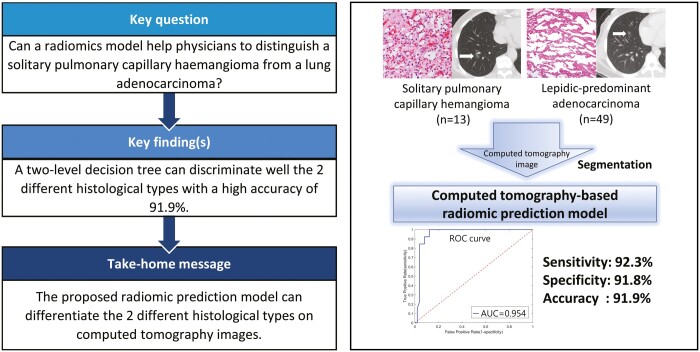

## INTRODUCTION

Solitary pulmonary capillary haemangioma (SPCH) is a primary benign lung tumour that exhibits proliferation of capillaries in the alveolar septa [1]. This uncommon disease was first described in a report of 8 autopsies in 2000 as ‘pulmonary capillary haemangiomatosis-like foci’ [2], and the term SPCH was first used in a report of 2 surgically resected patients in 2006 [3]. Only 17 cases of surgically resected SPCH have been reported in the English literature before 2018 [1, 3–6]. Clinically, SPCH usually presents as a ground-glass nodule (GGN) on chest-computed tomography (CT) images, which is similar to the presentation of lepidic-predominant adenocarcinoma (LPA) of the lung [1, 3–7].

Recently, the NELSON trial showed that lung cancer mortality is significantly lower among high-risk persons who underwent volume CT screening than among those who did not undergo screening [8]. Volume CT screening also enabled a significant reduction of harms, including positive tests and unnecessary workup procedures [8]. CT has been accepted as an effective lung cancer screening tool for high-risk patients [8, 9]. In ∼20% of the screened population, the screening detected indeterminate lung nodules; these patients may need further management [10]. In those patients, it is not uncommon that surgical resection reveals such lung nodules to be benign lung tumours, including SPCH. Recently, more cases of surgically resected SPCH have been reported [6, 7]. In 2018, Hsieh *et al.* [6] described a series of 16 surgically resected SPCH patients. All SPCHs were incidentally detected by CT screening, and all 16 SPCH lesions were initially unrecognized or misdiagnosed by general pathologists. Their report showed that SPCH may be underrecognized by radiologists in CT scans due to its similarity to early lung cancer and may also be underrecognized by general pathologists.

To assist in the identification of SPCH in thoracic CT images, this study aimed to extract radiomic features that can preoperatively distinguish SPCH from LPA. Although numerous radiomic features have been developed for the differential diagnosis of lung nodules [11], the differentiation of SPCH from LPA remains a difficult task because both types of lesions have very similar GGN-like appearances. Moreover, the limited number of cases reported for SPCH makes the training dataset intrinsically imbalanced. To overcome these difficulties, here, a radiomic analysis rooted in a divide-and-conquer paradigm is proposed to construct a two-level radiomic feature set as the basis of separating SPCH from LPA.

## MATERIALS AND METHODS

### Study population

**Figure 1:**
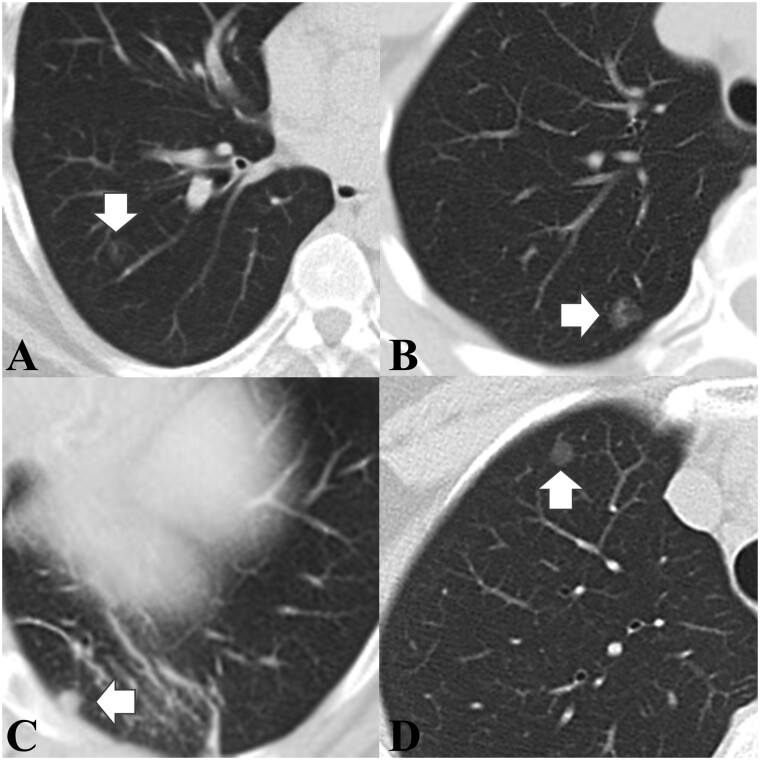
(**A and B**) Computed tomography (CT) image of solitary pulmonary capillary haemangioma (arrow), which usually presents as (**A**) a pure ground-glass nodule or (**B**) part-solid ground-glass nodule similar to the presentation of lepidic-predominant adenocarcinoma of the lung (arrow). (**C**) Occasionally, solitary pulmonary capillary haemangioma may present as a pure solid nodule. (**D**) CT image of lepidic-predominant adenocarcinoma (arrow).

Twenty-nine consecutive SPCH cases with tumour size >5 mm were considered in this study. These SPCH cases underwent surgical tumour resection by a single surgical team, using the same clinical protocols and perioperative orders, at the National Taiwan University Hospital between January 2013 and December 2017. Out of these 29 cases, 9 cases with other lung nodules in the same lobe were excluded from this study. Another 7 cases without preoperative thin-cut CT images were also excluded. Finally, 13 cases were enrolled in the SPCH group for further analysis (Fig. 1A). For the patient selection in the LPA group, we retrospectively evaluated 3327 consecutive patients who underwent thoracoscopic surgery for lung cancer by the same surgical team at our institute between January 2013 and December 2018. The inclusion criteria in the LPA group were as follows: (i) diagnosed as lung adenocarcinoma with pathologically confirmed near-pure (≥70%) lepidic-predominant histological subtypes and (ii) existence of preoperative thin-cut CT images. Finally, 49 cases were enrolled in the LPA group for further analysis (Fig. 1B).

**Figure 2:**
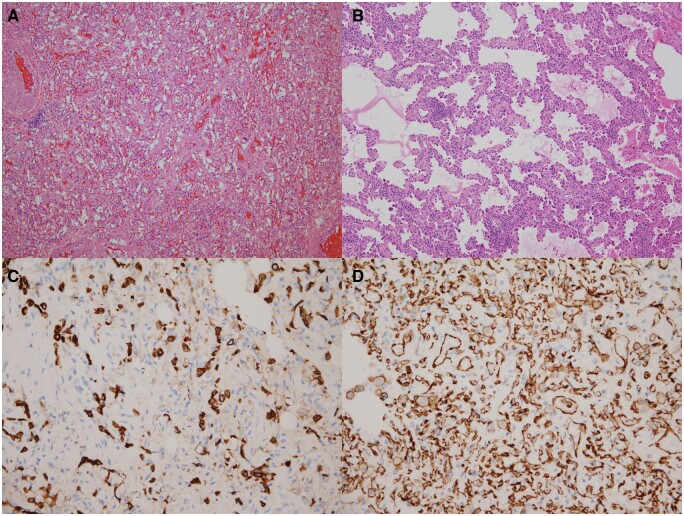
(**A**) Solitary pulmonary capillary haemangioma (SPCH) is characterized by the proliferation of capillary-sized vasculature. (**B**) Lepidic-predominant adenocarcinoma has neoplastic pneumocytes with large hyperchromatic nuclei growing along the alveolar walls. (**C**) Unlike lepidic-predominant adenocarcinoma, SPCH typically has decreased cytokeratin staining. (**D**) CD31 immunohistochemical staining highlights the proliferation of capillaries in SPCH [original magnification: (A** and B**) ×100; (C** and D**) ×400].

All pathological slides of the enrolled patients were reviewed according to the 2015 World Health Organization criteria [12]. The diagnosis of SPCH was made when the lesion size was >5 mm with no evidence of inflammation or adenocarcinoma in the submitted specimen, but decreased cytokeratin staining and increased CD31-positive vascular channels in immunohistochemical stainings (Fig. 2). The SPCH diagnosis and the percentage of the lepidic subtype were confirmed microscopically by an experienced thoracic pathologist (M.-S.H.).

### Image acquisition

The assessed CT images were the most recent CT images before surgery. All CT images in the DICOM format, without preprocessing or normalization, were considered. Chest CT images were obtained with a 16-, 64-, 128- or 256-detector row CT scanner from the following manufacturers: GE (LightSpeed 16, LightSpeed VCT, Revolution CT and Revolution RVO), Siemens (Emotion 16, Sensation 64 and SOMATON Definition AS+), Philips (iCT 256 and Ingenuity CT) and Canon (Aquilion ONE) Medical Systems. The CT image parameters were as follows: detector collimation, 0.5–0.625 mm; pitch, 0.813–1.2; gantry speed, 0.35 or 0.5 s per rotation; 120 kVp; 41–330 mA; slice thickness, 1.0–1.25 mm; and matrix, 512 × 512.

### Solitary pulmonary capillary haemangioma–lepidic-predominant adenocarcinoma classification model

The SPCH–LPA classification model was based on a divide-and-conquer radiomic analysis. The kernel idea was to untangle the intervened radiomic distribution of SPCH and LPA by 2 different sets of radiomic features. The first set partitioned the SPCH/LPA dataset into 2 subsets, including 1 with high confidence of being LPA and the other a mixture of SPCH and LPA to be further classified using a second set of radiomic features. The rationale behind this idea was to decompose the LPA samples into 2 subgroups, each of which was expected to have a higher homogeneity than its parent group. It reduced the originally imbalanced and intertwining classification problem into a relatively balanced problem with a more homogeneous subset of LPA, opening up an opportunity for better discrimination between SPCH and LPA.

**Figure 3:**
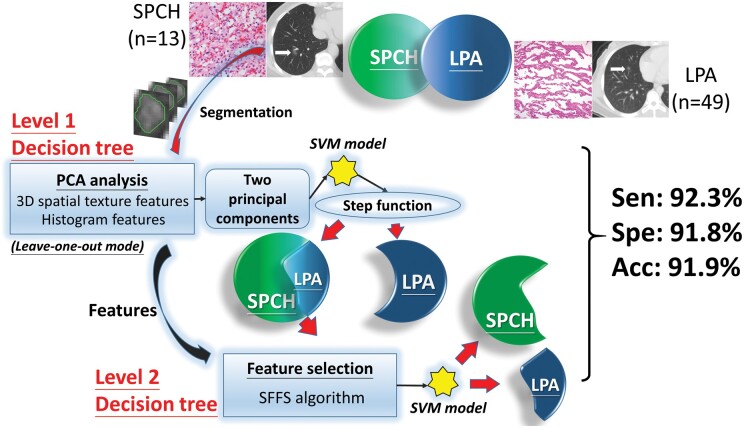
The schema of this study. LPA: lepidic-predominant adenocarcinoma; PCA: principal components analysis; SFFS: sequential forward feature selection; SPCH: solitary pulmonary capillary haemangioma; SVM: support vector machine.

The schema of this study is depicted in Fig. 3. The SPCH- or LPA-containing volumes of interest were first extracted from 3-dimensional (3D) thoracic CT images followed by segmentation processes demarcating the lesion boundaries. Texture features were extracted from histograms and the grey-level co-occurrence matrix (GLCM) [13] of the lesions with a divide-and-conquer paradigm. The performance of the proposed SPCH–LPA classification model was assessed using a leave-one-out cross-validation method.

### Tumour segmentation

The SPCH and LPA lesions were segmented semiautomatically with a level-set algorithm [14]. To further account for the complex compositions of SPCH and LPA, especially along the lesion boundaries, the computer-generated lesion boundaries were examined and, if necessary, modified manually by 2 chest radiologists (Y.-C.Chen and Y.-C.Chang) to reach consensus segmentation results. The tumour segmentation is described in detail in the Supplementary Methods.

### Feature extraction

To characterize SPCH and LPA, 2 types of radiomic features were extracted from the segmented lesions, namely the histogram features and the 3D spatial texture features. The histogram features included skewness, kurtosis, 75th percentile, 97.5th percentile and uniformity [15]. The 3D spatial texture features were composed of 21 features derived from the GLCM of each lesion. The histogram features characterized the grey-level distribution of a lesion, whereas the 3D spatial texture features described the spatial distribution of the grey levels within a lesion. More precisely, the 21 GLCM-based texture features modelled the grey-level co-occurrence characteristics of all horizontally adjacent voxels in a lesion.

### Feature selection and classification model building

An SPCH–LPA classification model was developed based on a two-level decision tree and 26 radiomic features extracted from each segmented lesion, including 5 and 21 features from the histogram and co-occurrence matrix, respectively. The two-level decision tree was constructed based on the training data with a support vector machine as the classifier in each tree node. For comparison, a baseline model was built with the same 26 features using a support vector machine as the classifier. The details of the feature selection and classification model building are provided in the Supplementary Methods.

### Performance assessment

To evaluate the performance of the proposed classification model, a leave-one-out cross-validation approach [16] was used in this study to estimate the model’s ability in predicting the new data that were not involved in the model construction. The details of the performance assessment are described in the Supplementary Methods.

### Statistical analysis

To investigate the differentiation capability of each radiomic feature, the independent two-sample *t*-test was conducted for each of the 26 features. Levene’s test was performed prior to the t-test to assess the homogeneity of variance of each radiomic feature with the null hypothesis of equal population variances. The significance levels of the independent two-sample *t*-test and Levene’s test were both set to 0.05. If the *P*-value of Levene’s test was less than the significance level, the null hypothesis was rejected and both groups, i.e. SPCH and LPA, were considered to have unequal variances for the tested radiomic feature. Otherwise, the group variances of SPCH and LPA were considered as equal. Receiver operating characteristic (ROC) curve analyses were carried out to assess the leave-one-out cross-validation performances of the proposed SPCH–LPA classification model and the baseline model using the probability of being an SPCH, i.e. *P*.

### Ethics statement and data availability statement

The Research Ethics Committee at our institute reviewed and approved this study (approval no. 202003074RIND; approval date, 14 April 2020), and the requirement for written informed consent was waived by the Institutional Review Board. All relevant data are within the manuscript and its Supporting Information files.

## RESULTS

### Demographic and clinicopathological characteristics

The characteristics of the 13 SPCH patients and 49 LPA patients investigated in our study are summarized in Table 1. In the SPCH group, a predominance of females (53.8%) and non-smokers (92.3%) was observed. The mean tumour diameter was 10.1 mm. The CT findings showed pure GGN, part-solid GGN and pure solid nodule in 4 (30.8%), 8 (61.5%) and 1 (7.7%) patients, respectively. Similar to the SPCH group, female (71.4%) and non-smoker (89.8%) predominance was also observed in the LPA group. In this group, most tumours were classified as pure GGN (71.4%) in CT images. The pathological features revealed non-invasive characteristics in most cases of the LPA group. The lepidic percentages were higher than 70% in all cases of the LPA group. Besides, neither micropapillary nor solid subtypes were noted in the enrolled patients.

**Table 1:** Clinicopathological features of the study cohort

	SPCH	LPA
Number of patients	13	49
Age (years)	54.5 ± 12.6 (38–77)	54.6 ± 11.4 (24–75)
Female	7 (53.8)	35 (71.4)
Non-smoker	12 (92.3)	44 (89.8)
Lung cancer family history	4 (30.8)	13 (26.5)
Abnormal CEA	1 (7.7)	0 (0)
Tumour location		
LUL	1 (7.7)	14 (28.6)
LLL	4 (30.8)	8 (16.3)
RUL	4 (30.8)	20 (40.8)
RML	1 (7.7)	3 (6.1)
RLL	3 (23.1)	4 (8.2)
Tumour diameter in CT image (mm)	10.1 ± 3.8 (6.0–19.0)	11.2 ± 5.9 (5.0–28.9)
Pathological tumour diameter (mm)	8.1 ± 3.9 (4.0–18.0)	9.1 ± 4.8 (4.0–25.0)
CT findings		
Pure GGN	4 (30.8)	35 (71.4)
Part-solid GGN	8 (61.5)	14 (28.6)
Solid	1 (7.7)	0 (0)
Tumour differentiation		
Well		43 (87.8)
Moderate		6 (12.2)
Visceral pleural invasion		0 (0)
Lymphovascular invasion		0 (0)
Lepidic percentage		
70–80%		8 (16.3)
80–90%		2 (4.1)
90–100%		22 (44.9)
Pure lepidic		16 (32.7)
pT stage		
AIS		15 (30.6)
T1mi		14 (28.6)
T1a		17 (34.7)
T1b		3 (6.1)
Lymph node metastasis		0 (0)

Data are presented as the mean ± SD or *n* (%). TNM staging is based on the eighth edition of the TNM classification.

CEA: carcinoembryonic antigen; CT: computed tomography; GGN: ground-glass nodule; LLL: left lower lobe; LPA: lepidic-predominant adenocarcinoma; LUL: left upper lobe; RLL: right lower lobe; RML: right middle lobe; RUL: right upper lobe; SPCH: solitary pulmonary capillary haemangioma; TNM: tumour, node and metastasis.

### Analysis of radiomic features

The radiomic feature analysis involved histogram features and 3D spatial texture features. The mean values of 7 features including kurtosis (*P* = 0.026), uniformity (*P* < 0.001), autocorrelation (*P* < 0.001), correlation (*P* < 0.001), sum of squares: variance (*P* < 0.001), sum average (*P* < 0.001) and sum variance (*P* < 0.001) were significantly different between SPCH and LPA. The detailed statistical analyses of the histogram features and the 3D spatial texture features are listed in Table 2.

**Table 2:** Histogram and 3D spatial texture feature analyses for patients with SPCH and LPA

	SPCH	LPA	*P*-value
Numbers of patients	13	49	
Histogram features			
Skewness	1.413 ± 0.773	1.227 ± 0.511	0.302
Kurtosis	4.888 ± 3.70	3.406 ± 1.415	0.026
75th percentile	87.75 ± 33.976	80.505 ± 30.563	0.461
97.5th percentile	128.55 ± 54.810	100.971 ± 35.913	0.106
Uniformity	0.0173 ± 0.005	0.029 ± 0.011	<0.001
Tumour region feature analysis by GLCM			
Autocorrelation	54.450 ± 43.098	215.698 ± 186.057	<0.001
Contrast	5.179 ± 4.420	3.667 ± 3.529	0.198
Correlation	0.501 ± 0.091	0.636 ± 0.121	<0.001
Cluster prominence	973.146 ± 982.489	2051.079 ± 5967.805	0.521
Cluster shade	−8.520 ± 60.754	62.069 ± 168.781	0.145
Dissimilarity	1.546 ± 0.742	1.255 ± 0.719	0.202
Energy	0.049 ± 0.026	0.066 ± 0.062	0.141
Entropy	3.512 ± 0.579	3.470 ± 0.955	0.844
Inverse difference	0.554 ± 0.099	0.603 ± 0.136	0.230
Inverse difference moment	0.505 ± 0.121	0.563 ± 0.165	0.242
Maximum probability	0.114 ± 0.044	0.127 ± 0.109	0.518
Sum of squares: variance	57.70 ± 45.249	216.875 ± 186.892	<0.001
Sum average	13.245 ± 5.961	25.005 ± 15.119	<0.001
Sum variance	160.969 ± 142.297	735.150 ± 651.642	<0.001
Sum entropy	2.481 ± 0.347	2.492 ± 0.526	0.930
Difference variance	5.179 ± 4.420	3.667 ± 3.529	0.198
Difference entropy	1.487 ± 0.341	1.306 ± 0.415	0.153
Information measure of correlation 1	−0.170 ± 0.052	−0.197 ± 0.075	0.234
Information measure of correlation 2	0.663 ± 0.099	0.685 ± 0.106	0.507
Inverse difference normalized	0.994 ± 0.003	0.995 ± 0.003	0.202
Inverse difference moment normalized	0.999 ± 0.00007	0.999 ± 0.00005	0.202

Data are presented as the mean ± SD.

3D: 3-dimensional; GLCM: grey-level co-occurrence matrix; LPA: lepidic-predominant adenocarcinoma; SD: standard deviation; SPCH: solitary pulmonary capillary haemangioma.

### Performance of the solitary pulmonary capillary haemangioma–lepidic-predominant adenocarcinoma classification model

For the root node of the two-level decision tree, 2 principal components corresponding to the 2 largest eigenvalues were extracted from the training data in each fold of the leave-one-out cross-validation process. The results of this process showed that the 2 principal components, on average, explained 99.88% ± 0.61% of the total variance of all training data in the 26-dimensional feature space in every fold. For the leaf node, only 4 texture features were selected, namely correlation, inverse difference, uniformity and information measure of correlation 2, which accounted for 61, 37, 14 and 12 times of feature selection, respectively, amounting to all 124 times of feature selection in the 62 folds of training processes.

**Figure 4:**
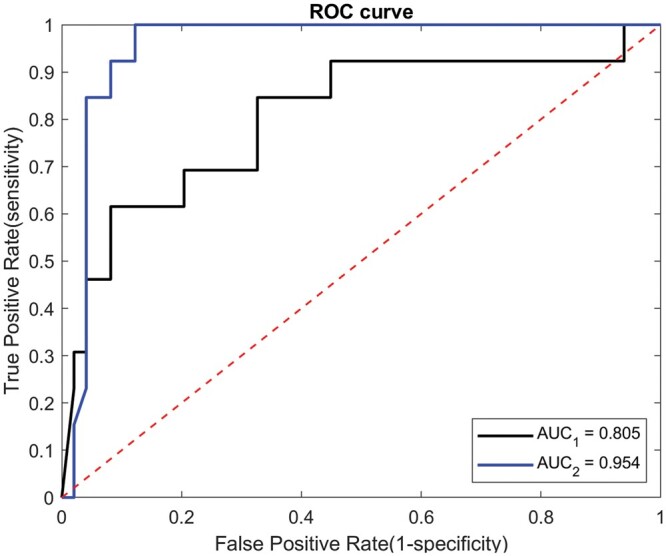
The receiver operating characteristic curve of the proposed solitary pulmonary capillary haemangioma/lepidic-predominant adenocarcinoma classification model (AUC_2_ = 0.954) and baseline model (AUC_1_ = 0.805). AUC: area under the curve; ROC: receiver operating characteristic.

The ROC curve of the proposed SPCH–LPA classification model is illustrated in Fig. 4, which was plotted based on the SPCH probability of each test datum, i.e. ${P=P}_{1}\times P_{2}$. The area under the curve (AUC) of the ROC curve was 0.954. The classification accuracy, sensitivity and specificity values of the proposed SPCH–LPA classification model were 91.9%, 92.3% and 91.8%, respectively, when a decision threshold of 0.5 was applied to the SPCH probability *P* (Supplementary Material, Table S1).

### Performance of the baseline model

Three to 6 features were selected by the sequential forward feature selection algorithm for each of the 62 folds in the leave-one-out cross-validation process as listed in Supplementary Material, Table S2, adding up to 285 times of feature selection in total. The total numbers of selections for each feature are summarized in Supplementary Material, Table S3. The 6 most frequently selected features were uniformity, correlation, dissimilarity, autocorrelation, energy and inverse difference, which were selected 62, 62, 35, 20, 18 and 17 times, respectively, representing 75.1% of feature selection in the 62 folds of training processes.

Figure 4 also displays the ROC curve of the baseline model with an AUC of 0.805. The classification accuracy, sensitivity and specificity values of this baseline model were 85.5%, 61.5% and 91.8%, respectively, for the decision threshold of 0.5 (Supplementary Material, Table S1).

To compare the differences between the AUC of the proposed SPCH–LPA classification model and the baseline model, a statistical method by Hanley and McNeil [17] was employed in this study to analyse differences between areas under 2 ROC curves. The AUC of our proposed SPCH–LPA classification model was significantly different from that of the baseline model (*P* = 0.025).

## DISCUSSION

Advances in CT imaging and the availability of CT screening programmes for lung cancer detection in asymptomatic high-risk patients have increased the detection rate of small pulmonary nodules [9, 10]. There are several guidelines and recommendations for the management and follow-up of incidentally encountered lung nodules detected by CT in adult patients [18, 19]. According to these recommendations, some indeterminate lung nodules are indicated for surgical resection. However, the final diagnosis of these indeterminate lung nodules includes not only malignancies but also benign lung tumours [10]. SPCH has been an uncommon benign lung tumour, with only 17 reported cases of surgically resected SPCH before 2018 [1, 3–6]. However, 25 more cases have been reported in the last 2 years [6, 7]. The increasing incidence of this benign tumour raises the importance of its preoperative diagnosis using imaging modalities. However, SPCH is characterized by GGNs in CT images and mimics LPA radiographically [1, 3–7]. Even experienced thoracic surgeons and radiologists may regard SPCH as early primary lung cancer. Therefore, it is important to differentiate SPCH from LPA by radiomic feature analysis. Our results showed that a radiomic texture feature-based classification model could differentiate SPCH from LPA on CT images.

Radiomic texture features have been widely used in tissue characterization for discriminating different types of lesions in thoracic CT images, such as automatic lung nodule detection [20–22], differential diagnosis of benign and malignant lung nodules [9, 10] and differentiation of lung cancer subtypes [11]. Several texture features have been suggested for automated detection of pulmonary nodules in CT images, including the mean, skewness and kurtosis values of intensity histograms [20, 21], local binary patterns [8, 22] and GLCM-based features [20, 21]. While radiomic texture features were shown to be effective in distinguishing various types of lung nodules, discrimination between SPCH and LPA was intrinsically difficult due to their common GGN-like appearance. Moreover, this task was challenging because the limited number of available SPCH cases evidently made the study population highly imbalanced.

The difficulty caused by the common GGN-like appearance could be partially observed in the similar texture features of the enrolled SPCH and LPA cases. As listed in Table 2, only 7 out of 26 radiomic features showed significant differences between the mean values of SPCH and LPA for the 62 lesions examined in this study. What made the differentiation between SPCH and LPA even more difficult was that the coefficients of variation, i.e. (standard deviation/mean) × 100, of these 26 features were mostly very high (>30), suggesting a high heterogeneity among each of the SPCH and LPA cohorts. As a result, the baseline model could only achieve an AUC of 0.805 with a combination of 3–6 features.

To cope with the problems of appearance similarity, cohort heterogeneity and imbalanced samples, a divide-and-conquer radiomic analysis approach was proposed in this study. A two-level SPCH–LPA decision tree was employed with the central idea of partitioning the LPA samples into 2 subsets. One subset comprised lesions with high confidence of being an LPA, whereas the other was expected to contain LPA lesions with higher discriminability from SPCH lesions for the subset was more homogeneous than its parent sample set. This idea was realized at the root node of the employed two-level decision tree. Specifically, it was accomplished by using the first 2 principal components of the training data in the 26-dimensional feature space and optimizing a support vector machine that maximized the positive predictive value subject to the constraint of 100% sensitivity. A lesion that was not picked out at the root node, i.e. *P*_1 = 1, was forwarded to the second level of the decision tree to determine the probability of being an SPCH lesion.

To avoid overfitting of the classification model, only 2 features were selected in the leaf node based on the training data in each fold of the leave-one-out cross-validation process, following the suggestion given by Jain *et al.* [23]. Noticeably, only 4 texture features had been selected in the 62 folds; the 3 most selected features were correlation, inverse difference and uniformity, which amounted to 90.3% of feature selections. Interestingly, these 3 features corresponded to 3 of the 4 characteristic appearance features which characterized the intensity similarity of horizontally adjacent voxel pairs. More precisely, the intensities of horizontally adjacent voxel pairs of an LPA were inclined to be more similar to each other than those of an SPCH. The CT image of incorrectly classified case was provided in Supplementary Material, Fig. S1. The CT images of the incorrectly classified case show similar intensities of adjacent voxel pairs that mimic LPA (Supplementary Material, Fig. S1).

The divide-and-conquer radiomic analysis embedded in the two-level SPCH–LPA decision tree demonstrated greatly enhanced discernibility of SPCHs from LPAs in comparison to the baseline classification model. The baseline model represented a generic machine learning model using a single set of radiomic features, which was the same set of texture features as used in the proposed two-level SPCH–LPA classification model. The respective AUC and accuracy values were significantly improved from 0.805 and 85.5% in the baseline model to 0.954 and 91.9% in the SPCH–LPA classification model.

###  

This study had several limitations. The first limitation was that tumour heterogeneity exists in lung adenocarcinoma. We tried to include LPA cases with the lepidic subtype of >70% to eliminate histological heterogeneities among analysed tumours in the LPA group and to extract representative radiomic information of LPA lesions. Second, relatively small numbers of SPCH and LPA cases were enrolled in this study, restricting the ability to account for tumour heterogeneity and texture variation caused by differences in CT scanner models. Because an SPCH diagnosis is uncommon, the number of patients with SPCH will remain limited until more cases are reported. Although the number of enrolled LPA cases might be increased in various ways, this approach would be constrained by the limited number of patients with SPCH due to the persistent data imbalance problem. However, the patient cohort in this study is the largest SPCH cohort in the currently existing literature, and our results are valuable to thoracic surgeons and radiologists worldwide. The accountability limitation for texture variation might have been partially weakened by the multiple varieties of CT scanner models employed in this study, which collated data from 3 makers and 10 models. Lastly, this was a retrospective study and did not exactly represent the real-world setting. The result of this study could only be applied to distinguish between SPCH and LPA, but not to differentiate SPCH from other subtypes of lung adenocarcinoma. Further validation with a larger cohort in a multicentre study is necessary.

Our study showed that SPCH could be accurately differentiated from lung cancer on CT images by a radiomic texture feature-based discriminant model. Our results may help surgeons to preoperatively discriminate between patients with SPCH and LPA, thus avoiding unnecessary surgery for benign lung tumours.

## SUPPLEMENTARY MATERIAL

Supplementary material is available at *ICVTS* online.

## Funding

This work was supported by the Ministry of Science and Technology, Taiwan [MOST 107-2221-E-002-080-MY3] and the National Taiwan University Hospital, Taipei, Taiwan [NTUH109-S4659, MS419].

##  

**Conflict** **of interest:** none declared.

### Author contributions

**Hao-Jen Wang:** Data curation; Formal analysis; Investigation; Methodology; Writing—original draft. **Mong-Wei Lin:** Conceptualization; Data curation; Formal analysis; Investigation; Methodology; Writing—original draft. **Yi-Chang Chen:** Investigation; Methodology; Writing—review & editing. **Li-Wei Chen:** Formal analysis; Investigation; Writing—original draft. **Min-Shu Hsieh:** Conceptualization; Data curation; Methodology; Writing—original draft. **Shun-Mao Yang:** Data curation; Methodology; Writing—review & editing. **Ho-Feng Chen:** Data curation; Formal analysis; Writing—review & editing. **Chuan-Wei Wang:** Data curation; Formal analysis; Writing—review & editing. **Jin-Shing Chen:** Conceptualization; Supervision; Writing—review & editing. **Yeun-Chung Chang:** Conceptualization; Data curation; Supervision; Validation; Writing—review & editing. **Chung-Ming Chen:** Conceptualization; Data curation; Supervision; Validation; Writing—original draft.

### Reviewer information

Interactive CardioVascular and Thoracic Surgery thanks Giuseppe Cardillo, Georges Decker, Marcelo F. Jimenez and the other, anonymous reviewer(s) for their contribution to the peer review process of this article.

## Supplementary Material

ivab271_Supplementary_Data

## ABBREVIATIONS

3D: 3-dimensional
AUC: Area under the curve
GGN: Ground-glass nodule
GLCM: Grey-level co-occurrence matrix
LPA: Lepidic-predominant adenocarcinoma
ROC: Receiver operating characteristic
SPCH: Solitary pulmonary capillary haemangioma
