# Supplementary material for: A radiomics model can distinguish solitary pulmonary capillary haemangioma from lung adenocarcinoma
Source: Interact Cardiovasc Thorac Surg. 2021 Oct 14;34(3):369–77. doi: 10.1093/icvts/ivab271 (PMC8860424; doi:10.1093/icvts/ivab271)
Supplement: ivab271_Supplementary_Data [file ivab271_supplementary_data.zip › Supplementary Figure S1.docx]

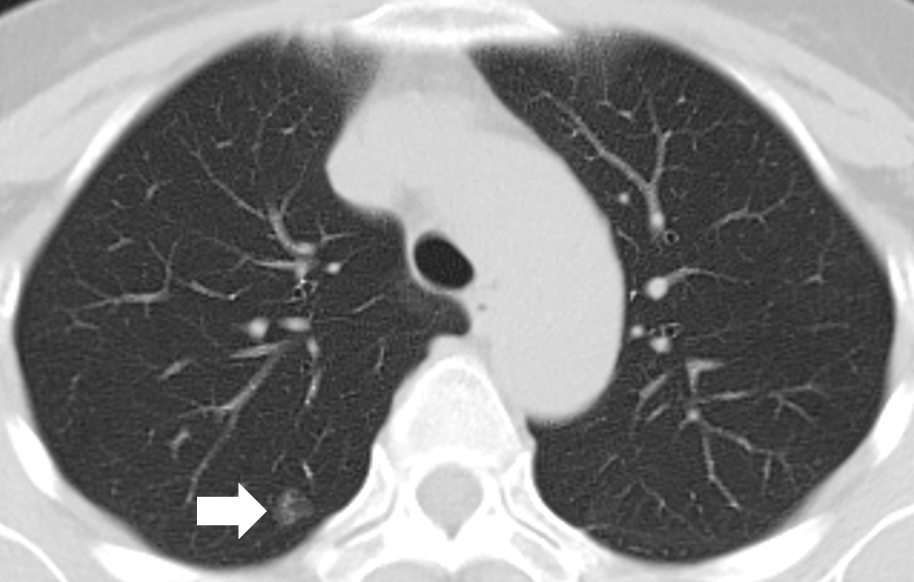


**Supplementary Figure S1**

Illustration showed CT image of incorrectly classified case that mimicked LPA.
