# Supplementary material for: A radiomics model can distinguish solitary pulmonary capillary haemangioma from lung adenocarcinoma
Source: Interact Cardiovasc Thorac Surg. 2021 Oct 14;34(3):369–77. doi: 10.1093/icvts/ivab271 (PMC8860424; doi:10.1093/icvts/ivab271)
Supplement: ivab271_Supplementary_Data [file ivab271_supplementary_data.zip › Supplementary Methods_revised.docx]

***Tumor segmentation***

Segmentation was a key step in the process of extracting radiographic features for the features were extracted from the lesion volumes defined by the derived lesion boundaries. However, the segmentation procedure was a challenging task for SPCH and LPA due to the weak lesion boundaries formed by the GGN-like appearance, as well as the complex structural compositions of blood vessels, bronchial tubes, pleural indentations, among others. To better describe the lesion boundaries of SPCH and LPA, a semiautomatic segmentation algorithm based on the hybrid level-set algorithm proposed by Zhang et al. was developed in the present study.^1^ The key ideas were to find a lesion boundary maximizing overall edge gradient strengths while ensuring regional uniformity within the boundary. To further account for the complex compositions of SPCH and LPA, especially, along the lesion boundaries, the computer-generated lesion boundaries were examined and, if necessary, modified manually by two chest radiologists (YC Chen and YC Chang) to reach consensus segmentation results.

***Feature selection and classification model building***

Based on the key idea of a divide-and-conquer radiomic analysis, the SPCH-LPA classification model, comprising a two-level decision tree with two support vector machines (SVMs), was employed to differentiate SPCH from LPA. The first level of the decision tree, i.e., the root node of the decision tree, was composed of an SVM followed by a step function, $u(p_{s1}-0.5)$, where $p_{s1}$ is the probability of a nodule being an SPCH estimated by the SVM with $u\left( x \right)=1$ for *x*≧0 and $u\left( x \right)=0$ for *x*<0. If we denote the probability output of a nodule at the root node of the decision tree by $p_{1}$, then $p_{1}=u(p_{s1}-0.5)$. The SVM of the root node was constructed based on the training data, the features of which comprised the first two principal components of the training data points in the feature space. The feature space was formed by the 26 features of all training data, including 5 histogram-based and 21 GLCM-based features. The first two principal components were the eigenvectors corresponding to the two largest eigenvalues derived via principal component analysis.^2^ The SVM of the root node maximized the positive predictive value subject to the constraint of 100% sensitivity. A true positive referred to the case that an SPCH was correctly classified as such.

Because the SVM of the root node was trained to have a sensitivity of 100%, the second level of the decision tree consisted of only one leaf node to further classify the positive outcomes of the root node into SPCH or LPA. The classifier of the leaf node was also an SVM but without being connected to a step function. The SVM of the leaf node was built using the 26 texture features. To avoid overfitting the classification model, a subset of features was selected to best differentiate SPCH from LPA by a sequential forward feature selection (SFFS) algorithm using the training dataset.^3^ While there is no unique rule in determining the maximum number of features for *n* training data, Jain et al. suggested that the number of features used for a two-class classification problem should be less than *n*/10.^4^ Considering the reduced number of training data at the leaf node, the number of features to be selected to train the SVM was set to 2 in this study. The SVM of the leaf node yielded the probability of a nodule being an SPCH, denoted by $p_{2}$. As a result, the probability of a test datum being classified as an SPCH would be ${p=p}_{1}p_{2}$. It should be noted that for a test datum with $p_{1}=0$, its probability of being an SPCH was set to 0, i.e., $p=0$.

***Performance assessment***

With a total of 62 cases, the leave-one-out cross-validation carried out 62 folds of cross-validation in a way that each case took turns to serve as the test data and the remaining 61 cases were treated as the training data. The training data were used not only to derive the two principal components for the root node and select the features for the leaf node but also to construct the two SVMs of the two-level decision tree.

To demonstrate the advantage of the divide-and-conquer radiomic analysis, a baseline classification model was implemented with the aim to separate SPCH from LPA using a single set of radiomic features. The baseline model used the SVM as the classifier with the same set of 26 texture features as the SPCH-LPA classification model. The performance of the baseline model was also evaluated by the leave-one-out cross-validation method. The SFFS algorithm was used to select the features in each fold using the training data. Following the rule suggested by Jain et al.^4^, the number of features selected in each fold was no more than 6.

References

1. Zhang Y, Matuszewski BJ, Shark LK, Moore CJ (2008) Medical image segmentation using new hybrid level-set method. In 2008 fifth international conference biomedical visualization: information visualization in medical and biomedical informatics,71-76. IEEE.
2. Mohanaiah P, Sathyanarayana P, GuruKumar L (2013) Image texture feature extraction using GLCM approach. International Journal of Scientific and Research Publications 3(5):1-5.
3. Ververidis D, Kotropoulos C (2008) Fast and accurate sequential floating forward feature selection with the Bayes classifier applied to speech emotion recognition. Signal Process 88:2956-2970.
4. Jain AK, Duin RPW, Mao J (2000) Statistical pattern recognition: A review. IEEE Trans Pattern Anal Mach Intell 22:4-37.
