# Supplementary material for: A radiomics model can distinguish solitary pulmonary capillary haemangioma from lung adenocarcinoma
Source: Interact Cardiovasc Thorac Surg. 2021 Oct 14;34(3):369–77. doi: 10.1093/icvts/ivab271 (PMC8860424; doi:10.1093/icvts/ivab271)
Supplement: ivab271_Supplementary_Data [file ivab271_supplementary_data.zip › Supplementary Table 1-3 revised.docx]

**Supplementary Table S1.** The classification accuracy, sensitivity, and specificity values of SPCH-LPA classification model and baseline model

**A.** SPCH-LPA classification model

|  | **AUC** | **Accuracy** | **Sensitivity** | **Specificity** |
| --- | --- | --- | --- | --- |
| **95% CI** | $0.954\pm0.049$ | $91.9\%\pm6.8\%$ | $92.3\%,\pm6.6\%$ | $91.8\%\pm6.8\%$ |

**B.** Baseline model

|  | **AUC** | **Accuracy** | **Sensitivity** | **Specificity** |
| --- | --- | --- | --- | --- |
| **95% CI** | $0.805\pm0.116$ | $85.5\%\pm6.8\%$ | $61.5\%,\pm12.1\%$ | $91.8\%\pm6.8\%$ |

Data are presented as the value ± 95% confidence interval.

Abbreviations: AUC, Area under the curve; CI, confidence interval; LPA, lepidic-predominant adenocarcinoma; SPCH, solitary pulmonary capillary hemangioma.

**Supplementary Table 1.** Features selected by the SFFS algorithm for each of the 62 folds in the leave-one-out cross-validation process

| **Fold** | **Selected feature 1** | **Selected feature 2** | **Selected**  **feature 3** | **Selected**  **feature 4** | **Selected**  **feature 5** | **Selected**  **feature 6** |
| --- | --- | --- | --- | --- | --- | --- |
| 1 | Uniformity | Correlation | Inverse difference normalized |  |  |  |
| 2 | Uniformity | Correlation | Inverse difference | Energy |  |  |
| 3 | Uniformity | Correlation | Inverse difference | Difference entropy |  |  |
| 4 | Uniformity | Correlation | Maximum probability | Energy |  |  |
| 5 | Uniformity | Correlation | Dissimilarity | Autocorrelation | Skewness | Sum of squares: Variance |
| 6 | Uniformity | Correlation | Dissimilarity | Autocorrelation | Cluster Shade |  |
| 7 | Uniformity | Correlation | Dissimilarity | Inverse difference | Sum variance |  |
| 8 | Uniformity | Correlation | Dissimilarity | Sum of squares: Variance | Skewness | Autocorrelation |
| 9 | Uniformity | Correlation | Inverse difference | Energy | Inverse difference moment normalized |  |
| 10 | Uniformity | Correlation | Dissimilarity | Inverse Difference Moment | Autocorrelation | Sum of squares: Variance |
| 11 | Uniformity | Correlation | Dissimilarity | Sum of squares: Variance | Energy |  |
| 12 | Uniformity | Correlation | Maximum probability | Energy | Sum average |  |
| 13 | Uniformity | Correlation | Contrast |  |  |  |
| 14 | Uniformity | Correlation | Inverse difference | Energy |  |  |
| 15 | Uniformity | Correlation | Contrast | Sum of squares: Variance | Autocorrelation |  |
| 16 | Uniformity | Correlation | Dissimilarity | Autocorrelation |  |  |
| 17 | Uniformity | Correlation | Inverse difference | Energy | Inverse Difference Moment |  |
| 18 | Uniformity | Correlation | Dissimilarity | Autocorrelation | Sum entropy |  |
| 19 | Uniformity | Correlation | Skewness | Inverse difference | Sum average |  |
| 20 | Uniformity | Correlation | Sum entropy | Sum average | Skewness |  |
| 21 | Uniformity | Correlation | Dissimilarity | Energy | Autocorrelation |  |
| 22 | Uniformity | Correlation | Dissimilarity | Autocorrelation | Inverse Difference Moment |  |
| 23 | Uniformity | Correlation | Dissimilarity | Energy |  |  |
| 24 | Uniformity | Correlation | Inverse difference | Energy | Inverse Difference Moment |  |
| 25 | Uniformity | Correlation | Dissimilarity | Sum of squares: Variance |  |  |
| 26 | Uniformity | Correlation | Inverse difference |  |  |  |
| 27 | Uniformity | Correlation | Dissimilarity | Sum variance | Difference entropy |  |
| 28 | Uniformity | Correlation | Dissimilarity |  |  |  |
| 29 | Uniformity | Correlation | Dissimilarity | Maximum probability |  |  |
| 30 | Uniformity | Correlation | Contrast | Sum variance | Skewness |  |
| 31 | Uniformity | Correlation | Inverse difference | Energy |  |  |
| 32 | Uniformity | Correlation | Dissimilarity | Energy |  |  |
| 33 | Uniformity | Correlation | Inverse difference | Energy |  |  |
| 34 | Uniformity | Correlation | Contrast | Autocorrelation | Cluster Shade |  |
| 35 | Uniformity | Correlation | Dissimilarity | Information measure of correlation2 |  |  |
| 36 | Uniformity | Correlation | Skewness |  |  |  |
| 37 | Uniformity | Correlation | Dissimilarity |  |  |  |
| 38 | Uniformity | Correlation | Contrast | Entropy | Sum average | Skewness |
| 39 | Uniformity | Correlation | Dissimilarity | Maximum probability | Autocorrelation |  |
| 40 | Uniformity | Correlation | Dissimilarity | Sum of squares: Variance |  |  |
| 41 | Uniformity | Correlation | Inverse difference | Energy | Inverse difference moment normalized |  |
| 42 | Uniformity | Correlation | Dissimilarity | Autocorrelation | Difference entropy |  |
| 43 | Uniformity | Correlation | Inverse difference | Energy | Inverse difference moment normalized |  |
| 44 | Uniformity | Correlation | Dissimilarity | Autocorrelation | Difference entropy |  |
| 45 | Uniformity | Correlation | Dissimilarity | Autocorrelation | Sum of squares: Variance |  |
| 46 | Uniformity | Correlation | Dissimilarity | Autocorrelation | Energy |  |
| 47 | Uniformity | Correlation | Dissimilarity |  |  |  |
| 48 | Uniformity | Correlation | Dissimilarity | Inverse difference |  |  |
| 49 | Uniformity | Correlation | Dissimilarity |  |  |  |
| 50 | Uniformity | Correlation | Skewness | Inverse difference | Sum average |  |
| 51 | Uniformity | Correlation | Dissimilarity | Autocorrelation | Skewness |  |
| 52 | Uniformity | Correlation | Inverse difference | Energy | Maximum probability |  |
| 53 | Uniformity | Correlation | Dissimilarity | Autocorrelation | Sum of squares: Variance | Sum average |
| 54 | Uniformity | Correlation | Skewness | Maximum probability |  |  |
| 55 | Uniformity | Correlation | Dissimilarity | Energy | Sum average | Skewness |
| 56 | Uniformity | Correlation | Contrast | Sum variance | 75^th^ percentile |  |
| 57 | Uniformity | Correlation | Dissimilarity | Inverse difference moment normalized | Inverse difference | Autocorrelation |
| 58 | Uniformity | Correlation | Dissimilarity | Autocorrelation | Sum of squares: Variance | 75^th^ percentile |
| 59 | Uniformity | Correlation | Dissimilarity | Autocorrelation | Entropy |  |
| 60 | Uniformity | Correlation | Dissimilarity | Sum variance |  |  |
| 61 | Uniformity | Correlation | Contrast | Sum average | Dissimilarity | Skewness |
| 62 | Uniformity | Correlation | Entropy |  |  |  |

Up to a maximum of six features were selected by the SFFS algorithm for each of the 62 folds in the leave-one-out cross-validation process. An early-stop occurred if the performance of validation data was not improved by further feature selection. Colors were used to represent the six most frequently selected features. Yellow represents the two most selected features, including uniformity and correlation. Both of them were selected for 62 times. Green, blue, purple and red represent the third, fourth, fifth and sixth most selected feature, respectively. Gray represents the other features which have fewer selected times than these six features.

**Supplementary Table 2.** Number of selections for each feature in the 62 folds of training processes

|  | **Number of selections** |
| --- | --- |
| **Histogram features** |  |
| Skewness | 12 |
| Kurtosis | 0 |
| 75^th^ percentile | 2 |
| 97.5^th^ percentile | 0 |
| Uniformity | **62** |
| **Tumor region feature analysis by GLCM** |  |
| Autocorrelation | **20** |
| Contrast | 7 |
| Correlation | **62** |
| Cluster Prominence | 0 |
| Cluster shade | 2 |
| Dissimilarity | **35** |
| Energy | **18** |
| Entropy | 3 |
| Inverse difference | **17** |
| Inverse Difference Moment | 4 |
| Maximum probability | 6 |
| Sum of squares: Variance | 10 |
| Sum average | 8 |
| Sum variance | 5 |
| Sum entropy | 2 |
| Difference variance | 0 |
| Difference entropy | 4 |
| Information measure of correlation 1 | 0 |
| Information measure of correlation 2 | 1 |
| Inverse difference normalized | 1 |
| Inverse difference moment normalized | 4 |
| **Total number of selections** | 285 |

Colors were used to represent the six most frequently selected features. Yellow represents the two most selected features, including uniformity and correlation. Both of them were selected for 62 times. Green, blue, purple and red represent the third, fourth, fifth and sixth most selected feature, respectively.
